# From Coffee Alkaloid to Ovarian Targets: An Integrated Computational Framework for Trigonelline in Ovarian Aging

**DOI:** 10.1002/fsn3.71421

**Published:** 2026-02-10

**Authors:** Woon Shin Yong, Yuankun Han, Yulu Shen, Ningyu Sun, Qinhua Zhang

**Affiliations:** ^1^ Department of Gynecology Shuguang Hospital Affiliated to Shanghai University of Traditional Chinese Medicine Shanghai China

**Keywords:** molecular docking, molecular dynamics, molecular pharmacology, network pharmacology, ovarian aging, trigonelline

## Abstract

Trigonelline, a coffee‐derived alkaloid with antioxidant and mitochondrial effects, has been proposed as a candidate modulator of aging, but its specific role in ovarian aging remains unclear. In this study, we applied an integrated in silico strategy combining network pharmacology, protein–protein interaction analyses, molecular docking, 100‐ns molecular dynamics simulations, and single‐cell transcriptomic data to identify potential trigonelline targets relevant to ovarian aging. Among 57 predicted targets, five (MMP9, JAK2, PARP1, HDAC1, and CYP3A4) emerged as core candidates spanning extracellular matrix remodeling, genome and epigenome maintenance, and endocrine/xenobiotic metabolism. Structural and molecular dynamics simulations combined with single‐cell expression patterns converged on PARP1 and MMP9 as the most plausible central mediators of trigonelline's putative age‐modulating effects in the ovary, with JAK2 and HDAC1 as intermediate candidates and CYP3A4 as a likely systemic mediator. These exploratory findings provide a conceptual framework that links coffee‐derived trigonelline to molecular pathways of ovarian aging and highlight specific targets and pathways for experimental validation in future nutrition and reproductive‐aging studies.

## Graphical abstract

This study employs an integrative in silico approach combining network pharmacology, molecular docking, and molecular dynamics simulations to explore the molecular association between trigonelline and ovarian aging, identifying five hub proteins (MMP9, JAK2, PARP1, HDAC1, and CYP3A4) and three major mechanistic axes related to extracellular matrix/inflammation, DNA–chromatin stress, and endocrine/xenobiotic regulation.

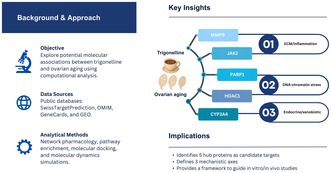

**Abbreviations**
∆G_bind_
: binding free energies
BP: biological process
CC: cellular component
CYP3A4: cytochrome P450 family 3 subfamily A member 4
GO: gene ontology
HDAC1: histone deacetylase 1
JAK2: janus kinase 2
KEGG: kyoto encyclopedia of genes and genomes
MD: molecular dynamics
MF: molecular function
MM/PBSA: molecular mechanics/Poisson‐Boltzman surface area
MMP9: matrix metallopeptidase 9
PARP1: poly(ADP‐ribose) polymerase 1
PPI: protein–protein interaction
Rg: radius of gyration
RMSD: root mean square deviation
SASA: solvent‐accessible surface area
UMAP: uniform manifold approximation and projection

## 1 Introduction

Extending ovarian function holds major implications for fertility, endocrine health, and long‐term cardiometabolic risk, and has therefore become a focus of efforts to identify tractable intervention points (Bochynska et al. 2025). Ovarian aging arises from interlinked processes, including extracellular matrix (ECM) remodeling and associated stromal fibrosis, chronic low‐grade inflammation, oxidative and mitochondrial stress, genomic instability, and disruption of steroid hormone production and metabolism (Zhu et al. 2025), yet the influence of food‐derived bioactive molecules on these aging processes remains insufficiently characterized.

Coffee ranks among the most extensively consumed dietary sources of bioactive compounds and correlates with modified aging trajectories (O'Keefe et al. 2018; Socała et al. 2020) and reproductive hormone profiles (Lucero et al. 2001; Sisti et al. 2015); however, most research has focused on caffeine. Coffee contains numerous bioactive metabolites, and among the major constituents, caffeine and chlorogenic acids have been extensively studied in relation to cardiometabolic and neurocognitive outcomes (Makiso et al. 2024; O'Keefe et al. 2018; Socała et al. 2020). By contrast, trigonelline, a small, polar alkaloid abundant in coffee and fenugreek, has more recently emerged as a coffee‐derived compound with distinctive mitochondrial and redox‐modulating actions at dietarily relevant concentrations (Nguyen et al. 2024; Membrez et al. 2024) and a favorable safety profile in human exposure and risk‐assessment studies (Konstantinidis et al. 2023), supported by its free‐radical‐scavenging, inflammation‐suppressing, antiglycative, and mitochondrial‐protective properties (Nguyen et al. 2024).

In a head‐to‐head human study, isolated trigonelline and chlorogenic acid both reduced early post–oral glucose tolerance test glucose and insulin responses compared with placebo (van Dijk et al. 2009), and metabolomic analyses consistently identify plasma or urinary trigonelline as one of the most robust biomarkers of habitual coffee intake, often outperforming caffeine and related metabolites (Guertin et al. 2015; Rothwell et al. 2014, 2019). Experimental studies further show that trigonelline modulates signaling and metabolism in several tissues (Nguyen et al. 2024), enhances cognition, and reduces neuroinflammation in animal models (Aktar et al. 2023; Chowdhury et al. 2018), and increases lifespan in *Caenorhabditis elegans* via activation of AMPK, DAF‐16, and HSF‐1 pathways (Zeng et al. 2021), suggesting broader relevance to geroscience.

Pharmacokinetic studies demonstrate that trigonelline is rapidly absorbed orally in humans, with peak plasma concentrations occurring 1–2 h post‐administration, alongside an elimination half‐life around 13–14 h (Mohamadi et al. 2021), indicating sustained systemic exposure. Animal studies further suggest that it crosses the blood–brain barrier (Farid et al. 2020) and vascular endothelium (Nguyen et al. 2024), implying broad tissue accessibility, potentially including the ovary. However, direct evidence describing trigonelline's role in ovarian biology remains sparse, and its molecular targets within the context of ovarian aging remain largely unexplored.

**FIGURE 1:**
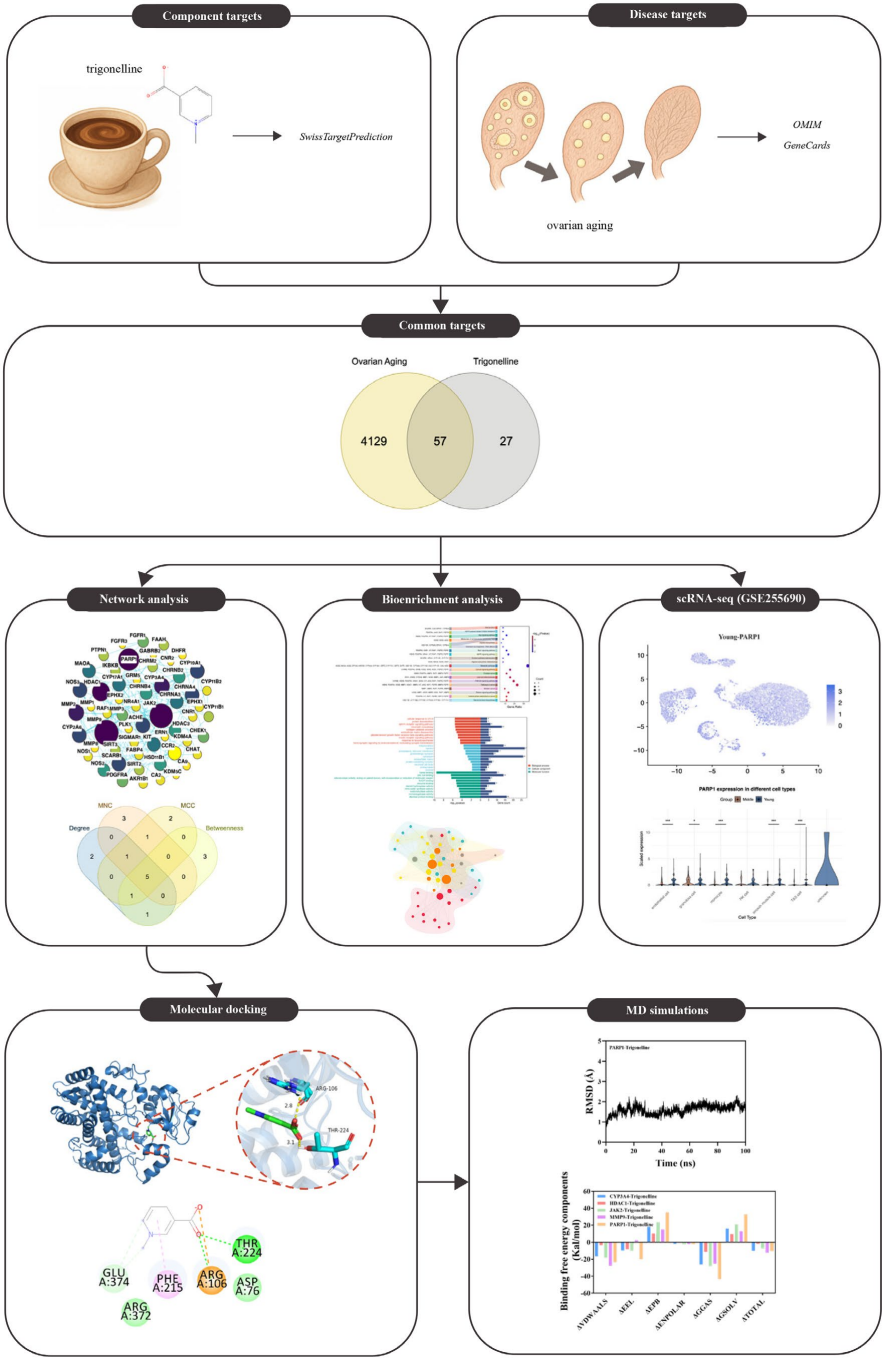
Schematic overview of the in silico workflow used to prioritize trigonelline targets in ovarian aging.

By integrating network pharmacology and computational simulations within a multi‐component, multi‐targeted, and multi‐pathway framework, this study addresses the existing gap by systematically predicting candidate targets and pathways of trigonelline's potential anti‐ovarian aging effects (Figure 1). It aims to provide a mechanistically informed foundation for subsequent experimental validation.

## 2 Materials and Methods

### 2.1 In Silico Target Identification for Trigonelline

Trigonelline's SMILES string (C[N+]1=CC=CC(=C1)C(=O)[O‐]) was obtained from PubChem (https://pubchem.ncbi.nlm.nih.gov/, accessed on February 5, 2025), with putative *Homo sapiens* protein targets predicted via SwissTargetPrediction (http://www.swisstargetprediction.ch/, accessed on February 5, 2025). The predicted targets were then deduplicated and normalized by gene symbol to compile a nonredundant trigonelline target list.

### 2.2 Cataloging Ovarian Aging‐Related Targets

Ovarian aging‐related targets were retrieved from OMIM (https://omim.org/, accessed on February 4, 2025) and GeneCards (https://www.genecards.org/, accessed on February 4, 2025) using the keyword “ovarian aging.” In GeneCards, genes with a relevance score ≥ 5 were retained to balance target coverage and specificity. After deduplication, the two databases yielded a consolidated list of ovarian‐aging associated targets.

### 2.3 Integration of Targets and Network Topology

Overlap between trigonelline and ovarian aging gene, identified through molbiotools, was uploaded to STRING (http://string‐db.org/, accessed on February 5, 2025), to yield a protein–protein interaction (PPI) network for *Homo sapiens* , with a minimum confidence threshold (0.4) and default settings. The network was subsequently exported to Cytoscape 3.10.4, and node centrality was assessed via the Cytohubba plugin. To improve robustness of hub selection, four centrality metrics (degree, betweenness, maximal clique centrality [MCC] and maximum neighborhood component [MNC]) (Chin et al. 2014) were calculated. For each metric, the top 10 genes were extracted, and genes that recurred across metrics were designated as robust hub candidates.

### 2.4 Pathway Enrichment Profiling

Intersecting targets were subjected to functional annotation of GO and KEGG using DAVID (https://davidbioinformatics.nih.gov/home.jsp, accessed on February 5, 2025). The *Homo sapiens* genome was used as the background and enrichment significance was defined as *p*‐value threshold of ≤ 0.05. GO terms were analyzed across three ontologies including Biological Process (BP), Cellular Component (CC), and Molecular Function (MF) to characterize the putative functional roles modulated by trigonelline. KEGG analysis mapped pathway perturbations linked to ovarian‐aging‐relevant genes of trigonelline. DAVID outputs were exported and reformatted to express enrichment as −log10(FDR). Visualizations were generated with an online tool (http://www.bioinformatics.com.cn, assessed on February 5, 2025) to present the top 10 GO terms per ontology and top 20 KEGG pathways ranked by −log10(FDR).

The same gene set was submitted to Reactome (https://reactome.org, accessed on November 25, 2025), and gene symbols were mapped to *Homo sapiens* pathways using the default background. Over‐represented pathways (FDR ≤ 0.05) were visualized in a ReacFoam map, in which enriched modules were grouped into higher‐order biological domains and colored by significance.

### 2.5 Single‐Cell Transcriptomic Analysis of Marker Gene Expression

GEO dataset GSE255690 (https://www.ncbi.nlm.nih.gov/geo/, accessed on November 15, 2025) provided single‐cell RNA sequencing data comprising ovarian samples of young (18–28 years) and middle‐aged (36–39 years) donors. Processed count matrices were analyzed with a Seurat (v5‐compatible) workflow using the RNA assay. Low‐quality cells were excluded based on standard filters for detected features, total counts, and mitochondrial read percentage, and genes detected in < 10 cells were removed. Data were log‐normalized (scale factor = 10,000), reduced by principal component analysis (PCA) and Uniform Manifold Approximation and Projection (UMAP), and clustered. Cell‐type annotations provided in the metadata were applied to assign clusters to major ovarian lineages and FeaturePlots were used for target gene visualization. For each gene–cell type–group stratum, summary statistics including mean, median, and the fraction of expressing cells (expression > 0) were calculated. Two‐sided Wilcoxon rank‐sum tests were applied to detect inter‐group differences across each gene–cell type combination, reporting *p*‐values and denoting significance in figures (*p* < 0.05). Although the dataset includes an additional “Old” cohort (47–49 years), these samples were excluded from the current analysis to focus on the clinically relevant reproductive window. This older cohort may be analyzed in future studies to explore extended aging trends.

### 2.6 Docking Protocol and Interaction Visualization

The 2D ligand structures were sourced from PubChem (http://pubchem.ncbi.nlm.nih.gov/, accessed on November 6, 2025) and converted to 3D conformations using ChemOffice, saved in MOL2 format. Protein targets were obtained from the RCSB PDB database (http://www.rcsb.org/, accessed on November 6, 2025), PDB IDs: 5VCC, 7SME, 8BXH, 6ESM, 6NRH, and high‐resolution crystal structures were selected as docking receptors. Water molecules and phosphate groups were removed in PyMOL 2.6. Structures were prepared for docking with AutoDock 1.5.6 by adding hydrogens, assigning rotatable bonds (torsions), and defining docking grids (Bitencourt‐Ferreira et al. 2019). Molecular docking was carried out using AutoDock Vina to evaluate possible protein–ligand interactions. For each target, the conformation with the lowest predicted binding energy was selected as the optimal pose (Wang et al. 2016). Interaction visuals were produced in Discovery Studio 2019 and PyMOL 2.6, with 2D and 3D residue‐level representations. In this scoring system, binding energies below −5.0 kcal/mol generally indicate moderate binding affinity, whereas values below −7.0 kcal/mol reflect strong predicted affinity and greater conformational stability. Lower (more negative) binding energies therefore correspond to stronger predicted protein–ligand binding.

### 2.7 Structural Stability and Dynamics via MD Simulations

Molecular dynamics (MD) simulations were carried out to assess complex stability using GROMACS 2022. Protein topologies were generated using the AMBER14SB force field via pdb2gmx, and ligand parameters were assigned using GAFF2 with RESP charges generated through Antechamber via the AutoFF interface. The complexes were embedded in a cubic TIP3P water box with a 1.0 nm buffer, neutralized and adjusted to 0.15 M NaCl with gmx genion. Electrostatic interactions were computed using the Particle Mesh Ewald (PME) method with a 1.0 nm cutoff, and van der Waals interactions employed the same cutoff. All covalent bonds involving hydrogen atoms were constrained with the LINCS algorithm, allowing an integration timestep of 2 fs (Mark and Nilsson 2001).

Energy minimization was first performed using steepest descent, followed by conjugate‐gradient optimization under restrained and unrestrained conditions. The system was equilibrated under the NPT ensemble, maintaining a temperature of 310 K using the Nosé–Hoover thermostat and a pressure of 1 bar with the Parrinello–Rahman barostat. Production MD runs lasted 100 ns, with trajectories recorded for subsequent analysis. Trajectory analyses included root mean square deviation (RMSD) to assess global structural stability, root mean square fluctuation (RMSF) to evaluate residue‐level flexibility, radius of gyration (Rg) to monitor overall compactness, hydrogen bonding to track ligand‐protein interactions, and solvent‐accessible surface area (SASA) to assess changes in solvent exposure, using standard GROMACS tools. Binding free energies were further calculated using MM‐PBSA via the g_mmpbsa package.

## 3 Results

### 3.1 Prediction of Trigonelline Targets and PPI Topology

**FIGURE 2:**
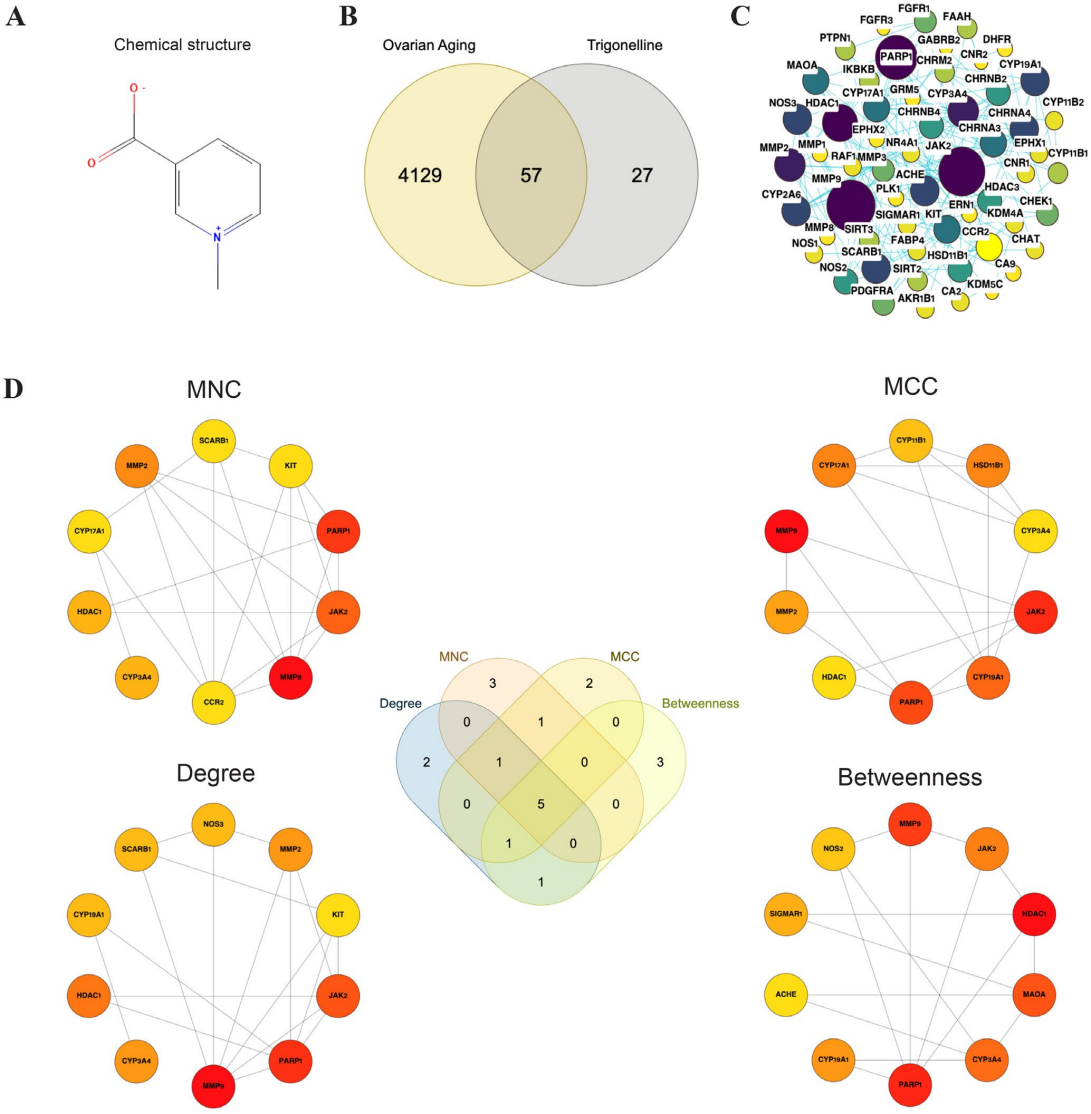
Chemical structure of trigonelline and identification of shared targets with ovarian aging–associated genes. (A) Two‐dimensional chemical structure of trigonelline. (B) Venn diagram showing the overlap (*n* = 57) between consolidated ovarian aging–associated genes (*n* = 4184) and predicted trigonelline targets (*n* = 84). (C) STRING protein–protein interaction (PPI) network of the 57 intersecting targets, with node size and color indicating degree centrality. (D) CytoHubba centrality analysis of the PPI network. The top 10 genes for each of four centrality metrics (degree, maximal clique centrality [MCC], maximum neighborhood component [MNC], and betweenness centrality) are displayed. Genes overlapping across all four rankings, MMP9, JAK2, PARP1, HDAC1, and CYP3A4, are designated as core hubs.

The chemical structure of trigonelline is depicted in Figure 2A. A total of 84 targets associated with trigonelline were predicted from the SwissTargetPrediction database. GeneCards and OMIM databases were queried for ovarian aging, yielding 4184 and 60 targets, respectively. After merging and removing duplicate gene symbols, a total of 4186 targets were obtained. A Venn diagram analysis was conducted to obtain the overlapping targets, pinpointing 57 shared genes as likely mediators in the ovarian aging effects of trigonelline (Figure 2B).

The PPI network for the 57 intersecting targets was established utilizing the STRING database and visualized through Cytoscape version 3.10.4. The network consisted of 57 nodes and 175 edges, with node characteristics (size and color intensity) reflecting connectivity (degree values) (Figure 2C). Using four Cytohubba metrics (degree, MCC, MNC, and betweenness centrality), the top 10 genes were identified (Figure 2D; Table S1). Five genes—MMP9, JAK2, PARP1, HDAC1, and CYP3A4—overlapped across all four metrics and were defined as robust core hubs (Figure 2D). These hubs suggest entry points where trigonelline may exert widespread regulatory influence on ovarian aging.

### 3.2 Functional Landscape of Shared Targets

**FIGURE 3:**
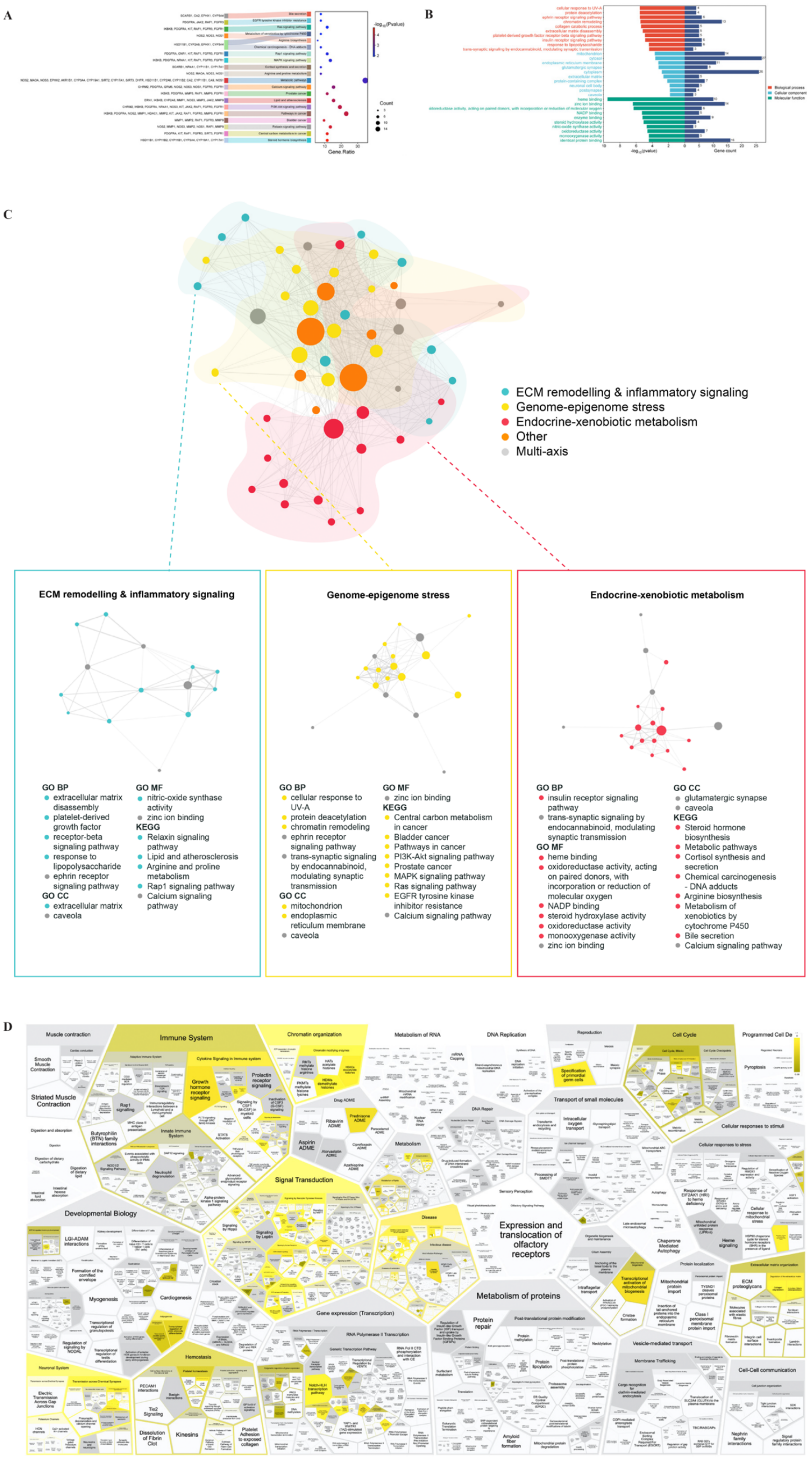
Integrated pathway enrichment and network analysis of trigonelline–ovarian aging targets. (A) Top 20 enriched KEGG pathways (*p* < 0.05) plotted by significance and gene count. (B) Top 10 enriched GO biological process, cellular component, and molecular function terms (*p* < 0.05) plotted by significance and gene count. (C) Gene–concept network of enriched KEGG and GO, with node size proportional to gene count, edge thickness reflecting shared genes, node color indicating mechanistic axis and darker gray nodes denoting multi‐axis terms. (D) Reactome ReacFoam map of pathway enrichment for trigonelline‐ovarian aging targets. Each polygon represents a Reactome pathway, grouped hierarchically into super‐pathways (e.g., immune system, signal transduction, ECM organization, mitochondrial function). Polygon area reflects pathway size (number of entities), and yellow tiles indicate significantly enriched pathways (*p* < 0.05).

Enrichment analyses of the 57 intersecting targets elucidated involvement in 33 KEGG signaling pathways (FDR‐adjusted *p* < 0.05; Figure 3A), notably steroid hormone biosynthesis (hsa00140), PI3K‐Akt signaling, relaxin signaling, lipid and atherosclerosis, and central carbon metabolism pathways, among others. GO terms annotated 152 BPs, 26 CCs, and 109 MFs. As illustrated in Figure 3B, in the BP category, the main enrichments were positive in cellular response to UV‐A, protein deacetylation, ephrin receptor signaling pathway, chromatin remodeling, collagen catabolic process. Highlighted CC terms were mitochondrion, cytosol, endoplasmic reticulum membrane, glutamatergic synapse, and cytoplasm while principal MF terms encompassed heme and zinc ion binding, oxidoreductase, NADP binding, and enzyme binding. These results converge on ECM remodeling and inflammatory signaling, endocrine and xenobiotics signaling, and chromatin dynamics (Figure 3C).

Reactome over‐representation analysis indicated that the shared genes were predominantly enriched in immune and cytokine signaling, extracellular matrix organization, chromatin and DNA replication/repair, and mitochondrial–metabolic pathways. On the ReacFoam map, significant pathways (yellow tiles) aggregated into coherent blocks corresponding to immune system signaling (including growth hormone and prolactin receptor signaling), ECM proteoglycans and extracellular matrix organization, chromatin organization and DNA repair, mitochondrial biogenesis and respiratory chain function, and heme‐ and xenobiotic‐related metabolism. These Reactome modules broadly mirrored the enrichment patterns seen in KEGG and GO, indicating that the gene set spans inflammatory/ECM, genome‐epigenome stress and endocrine‐xenobiotic metabolic processes relevant to ovarian aging.

### 3.3 Single‐Cell Transcriptomic Analysis of Candidate Targets in Human Ovarian Tissue

Employing UMAP dimensionality reduction and validated cell‐type markers, single‐cell transcriptome analysis of human ovarian samples from GSE255690 (young 18–28 years; middle aged 36–39 years) was partitioned into endothelial, granulosa, monocyte, natural killer (NK), smooth muscle, theca/stroma, and an unknown cluster. Among the five candidate targets, four genes (HDAC1, JAK2, MMP9, and PARP1) demonstrated statistically significant differences in age‐associated expression in at least one lineage (*p* < 0.05 for all, Section 2), whereas CYP3A4 showed minimal to no expression in the ovarian tissue and no evidence of age‐related alterations.

**FIGURE 4:**
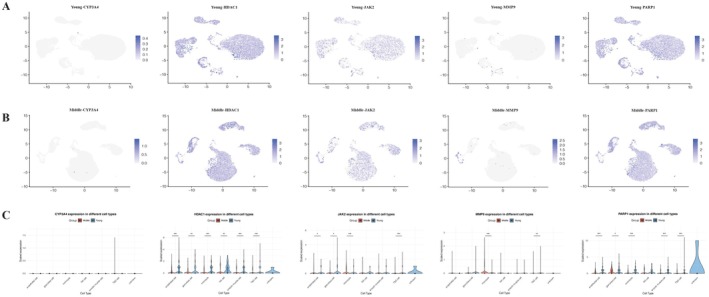
Single‐cell expression patterns of trigonelline hub targets across the aging ovary. (A) UMAP feature plots of MMP9, JAK2, PARP1, HDAC1, and CYP3A4 in young ovaries. (B) UMAP feature plots of the same genes in middle‐aged ovaries. In (A, B), color intensity reflects normalized expression per cell. (C) Violin plots of MMP9, JAK2, PARP1, HDAC1 and CYP3A4 expression by cell type and age group, illustrating broadly high, age‐sensitive HDAC1 expression, modest but widespread PARP1/JAK2 expression, niche‐specific MMP9 signal and minimal CYP3A4 transcription. Asterisks indicate statistically significant differences between young and middle‐aged groups within a given cell type. *p < 0.05, **p < 0.01, ***p < 0.001; ns, not significant.

Feature plots and violin plots (Figure 4A–F) revealed gene‐ and lineage‐specific transcriptional remodeling rather than a uniform pattern across the ovary. HDAC1 displayed the most widespread age‐related shift, with significantly lower expression in middle‐aged samples across endothelial, granulosa, monocyte, NK, smooth muscle, and theca/stroma clusters (all comparisons, *p* < 0.05). PARP1 expression also differed between age groups in several lineages, with significantly lower levels in middle‐aged endothelial, monocyte, smooth muscle, and theca/stroma cells but slightly higher expression in middle‐aged granulosa cells (all *p* < 0.05). JAK2 exhibited significant age‐related differences in endothelial, granulosa, monocyte, and theca/stroma populations (*p* < 0.05), with expression decreasing in middle‐aged endothelial, granulosa, and theca/stroma cells but increasing in monocytes, and did not reach significance in NK or smooth muscle cells. MMP9 expression was largely confined to monocytes where it was significantly higher in middle‐aged samples and theca/stroma cells, where it was low level but had significant downregulation (*p* < 0.05), while other lineages remained at or near the detection limit. CYP3A4 showed no discernible expression pattern and did not differ between age groups in any lineage. A summary of the directionality of the related change for the five hub genes across ovarian cell types is provided in Table 1.

**TABLE 1:** Summary of age‐related changes in hub gene expression in ovarian single‐cell clusters (middle‐aged vs. young, GSE255690).

Hub gene	Age‐related expression pattern (middle‐aged vs. young)	Functional interpretation in ovarian aging
HDAC1	↓^a^ across major lineages (endothelial, granulosa, monocyte, NK, smooth muscle, theca/stroma)	Reduced HDAC1 may promote epigenetic drift and impaired stress resilience in ovarian cells
PARP1	Generally ↓^a^ (endothelial, monocyte, theca/stroma, smooth muscle), slight ↑^a^ in granulosa	Lower PARP1 in stromal/vascular cells may weaken DNA repair, while granulosa upregulation may reflect compensatory stress responses
JAK2	↓^a^ in endothelial, granulosa, theca/stroma; ↑^a^ in monocytes	Decreased JAK2 in structural lineages may dampen cytokine/growth factor signaling; increased monocyte JAK2 may enhance inflammatory and redox signaling
MMP9	↑^a^ in monocytes; low‐level ↓^a^ in theca/stroma	Higher monocyte MMP9 is consistent with enhanced matrix turnover and inflammation, potentially favoring fibrotic remodeling
CYP3A4	ND (near‐zero expression in all lineages)	Lack of ovarian CYP3A4 expression supports a predominantly systemic role via hepatic steroid and xenobiotic metabolism

^a^ ↑ increased expression in middle‐aged vs. young; ↓ decreased; ND, not detected at the single‐cell level.

### 3.4 Verification of Molecular Docking

Molecular docking was used to predict the binding mode of trigonelline with the top five proteins identified from the PPI network: CYP3A4, HDAC1, JAK2, MMP9, and PARP1. Docking scores ranged from −4.6 to −6.5 kcal/mol (Table 1), indicating moderate binding affinity based on the applied scoring scheme. None of the proteins reached the high‐affinity range (≤ −7.0 kcal/mol). Among them, CYP3A4 (−6.5 kcal/mol), PARP1 (−6.2 kcal/mol), and MMP9 (−6.1 kcal/mol) displayed slightly higher binding affinities than JAK2 (−5.3 kcal/mol) and HDAC1 (−4.6 kcal/mol).

**FIGURE 5:**
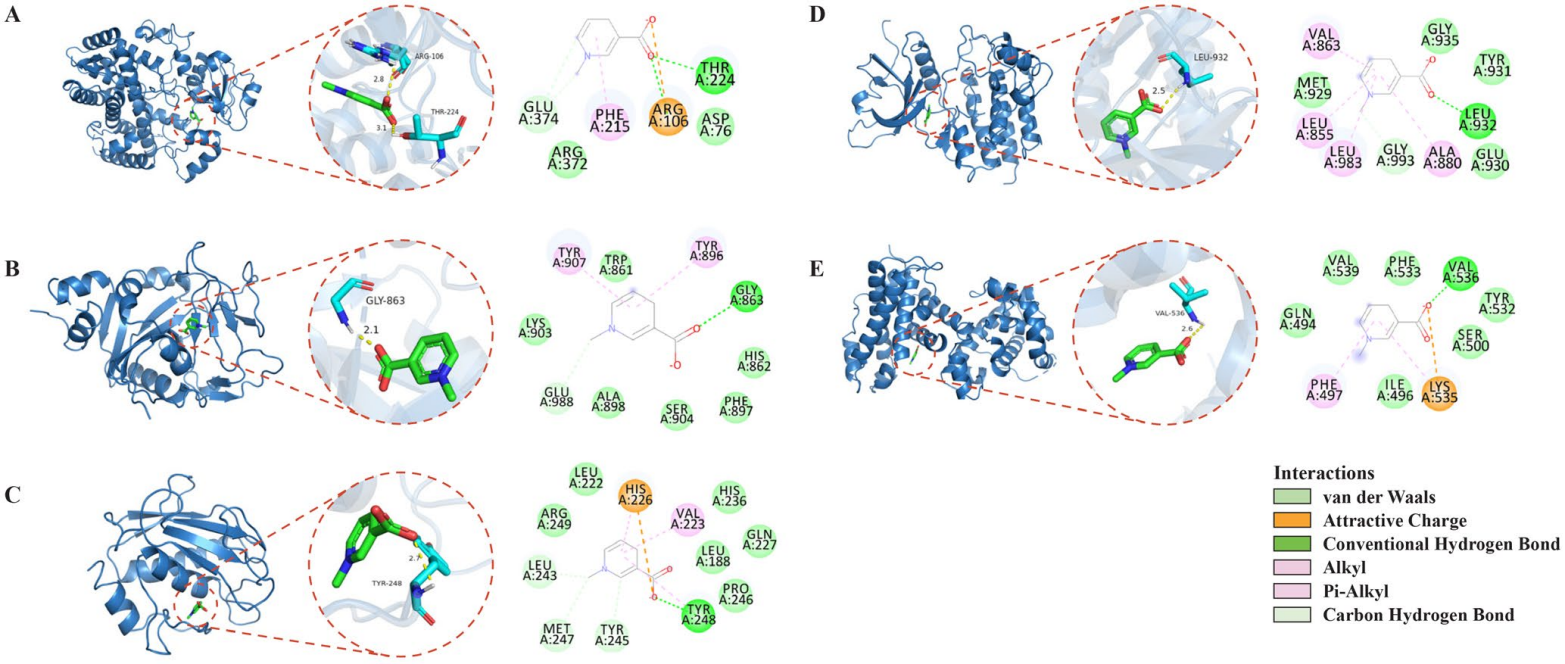
Molecular docking poses and interaction diagrams of trigonelline with hub proteins. (A) CYP3A4–trigonelline complex. Trigonelline occupies the distal substrate‐binding pocket, forming hydrogen bonds primarily with THR224 and ARG106, surrounded by hydrophobic and van der Waals contacts from nearby residues (e.g., PHE215, ASP76, ARG372, GLU374). (B) PARP1–trigonelline complex. Trigonelline occupies the ligand‐binding pocket, forming a hydrogen bond with GLY863, hydrophobic interactions with TYR896 and TYR907, van der Waals contacts with LYS903, ALA898, SER904, PHE897, HIS862, and TRP861, and a C—H bond with GLU988. (C) MMP9–trigonelline complex. The ligand binds within the catalytic pocket, engaging HIS226 via electrostatic attraction and TYR248 via hydrogen bonding. Hydrophobic interactions involve LEU222, TYR248, VAL223, and HIS226, supported by van der Waals contacts with ARG249, LEU222, HIS236, GLN227, LEU188, and PRO246, and C—H bonds with MET247, TYR245, and LEU243. (D) JAK2–trigonelline complex. Trigonelline is located in the ATP‐binding hinge region, forming a hydrogen bond with LEU932, hydrophobic interactions with LEU983, VAL863, ALA880 and LEU855, van der Waals contacts with GLY935, TYR931, GLU930 and MET929, and a C—H bond with GLY993. (E) HDAC1–trigonelline complex. The ligand interacts with VAL536 (hydrogen bonding) and PHE497 and LYS535 (hydrophobic and electrostatic contacts), supported by additional van der Waals interactions with nearby residues. Due to extended residue numbering in the HDAC1 structure used, the precise relationship of this pose to the canonical Zn^2+^‐containing catalytic center should be interpreted with caution.

For all targets, trigonelline adopted stable binding poses within the predicted binding pocket, stabilized through a combination of hydrogen bonding, van der Waals contacts, hydrophobic interactions, and in some complexes, additional electrostatic and carbon–hydrogen (C—H) bond interactions. In CYP3A4 (Figure 5A), trigonelline formed hydrogen bonds with THR224, ARG106, and hydrophobic interactions with PHE215, supported by van der Waals interactions with ASP76 and ARG372 and a C—H bond with GLU374 in the distal substrate‐binding pocket. For PARP1 (Figure 5B), trigonelline formed a hydrogen bond with GLY863, hydrophobic interactions with TYR896 and TYR907, and additional stabilizing van der Waals interactions with SER904 and HIS862 within the ligand‐binding pocket.

Docking analysis showed that in MMP9 (Figure 5C), trigonelline engaged HIS226 via electrostatic attraction and TYR248 via hydrogen bonding. Hydrophobic contacts were observed with TYR248, VAL223, and HIS226, supported by van der Waals contributed from ARG249, LEU222, HIS236, GLN227, LEU188, and PRO246. C—H bonds with MET247, TYR245, and LEU243 further contributed to ligand binding within the pocket. Other than that, trigonelline bound to JAK2 (Figure 5D) through a hydrogen bond with LEU932 and hydrophobic interactions with VAL863, LEU983, ALA880, and LEU855, and van der Waals contacts with GLY935, TYR931, GLU930, and MET929. A C—H bond with GLY993 also stabilized the ligand in the binding site. In the HDAC1 complex (Figure 5E), interactions were observed with VAL536, PHE497, and LYS535. Because the residue numbering of the HDAC1 structure used extends beyond the canonical length of HDAC1, assignment of this pose to the catalytic Zn^2+^ center should be interpreted with caution.

Overall, the docking results suggest that trigonelline can form plausible, moderately favorable interactions across multiple targets relevant to ovarian physiology and stress responses. CYP3A4, PARP1, and MMP9 exhibited the most favorable docking energies and extensive contact networks. Although none of the targets reached the high‐affinity threshold (≤ −7.0 kcal/mol), their spatial overlap with known functional sites strengthens the biological plausibility of the docking predictions.

### 3.5 MD Simulation Results

**FIGURE 6:**
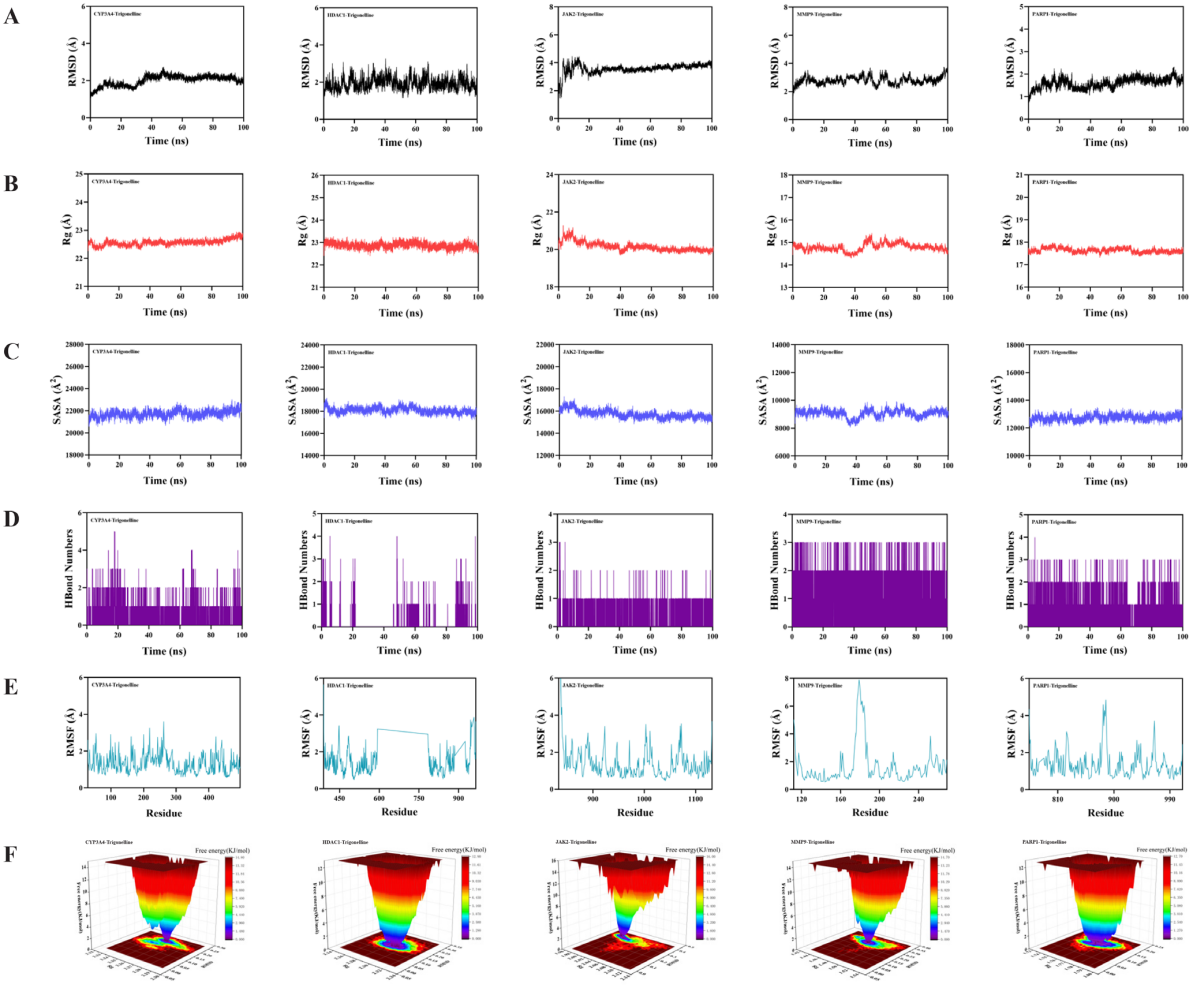
Molecular dynamics of trigonelline bound to CYP3A4, HDAC1, JAK2, MMP9, and PARP1. (A) Backbone RMSD profiles of each protein–ligand complex, showing equilibration and fluctuation ranges over time. (B) Radius of gyration (Rg) of each complex, indicating global compactness and absence of major expansion or collapse. (C) Solvent‐accessible surface area (SASA) trajectories, reflecting overall solvent exposure of the complexes. (D) Time evolution of hydrogen bonds between trigonelline and each protein, showing intermittent but recurrent contacts. (E) Per‐residue RMSF values for protein backbones, highlighting generally low flexibility at binding regions and a flexible loop around residues 180–200 in MMP9. (F) Free energy landscapes (FELs) constructed from RMSD and Rg, indicating dominant low‐energy basins and convergence to stable conformational states for all complexes.

We next evaluated the 100‐ns trajectories for each trigonelline‐protein complex to assess structural stability (Table 2). RMSD profiles showed all complexes remained within stable fluctuation ranges (Figure 6A). The CYP3A4 and PARP1 complexes displayed the lowest backbone deviations at 2.2 and 2.0 Å, respectively, followed by HDAC1–trigonelline at ~2.5 Å. The JAK2–trigonelline complex displayed slightly higher RMSD values, stabilizing near 3.8 Å, and the MMP9–trigonelline complex showed moderate fluctuations in the first half of the simulation and maintained an average RMSD of around 3.5 Å.

**TABLE 2:** Integrated summary of docking scores, MM/PBSA binding free energies and molecular dynamics metrics for trigonelline–target complexes.

Target protein	Docking score (kcal/mol)	MM/PBSA ΔGbind (kcal/mol)	RMSD Plateau (Å)	Typical H‐bond count
CYP3A4	−6.5	−9.97	~2.2	~2 (range 0–5)
PARP1	−6.2	−10.38	~2.0	~2 (range 0–4)
MMP9	−6.1	−12.28	~3.5	~2 (range 0–3)
JAK2	−5.3	−7.21	~3.8	~1 (range 0–3)
HDAC1	−4.6	−1.86	~2.5	~1 (range 0–4)

Abbreviations: ΔG_bind, binding free energy; MM/PBSA, molecular mechanics Poisson–Boltzmann surface area; RMSD, root mean square deviation.

Additionally, Rg (Figure 6B) and SASA (Figure 6C) trajectories exhibited only minor fluctuations for all systems, indicating that trigonelline did not induce major global expansion or collapse of protein structures. Moreover, hydrogen bond analyses revealed intermittent but recurrent hydrogen bonding with trigonelline throughout the simulation (Figure 6D). CYP3A4 complex formed 0–5 hydrogen bonds (typically 2), HDAC1 complex formed 0–4 hydrogen bonds (typically 1), JAK2 complex formed 0–3 hydrogen bonds (typically 1), MMP9 complex formed 0–3 hydrogen bonds (typically 2), and PARP1 complex formed 0–4 hydrogen bonds (typically 2). RMSF plots (Figure 6E) further confirmed low backbone dynamics across most residues, except for a distinct peak in MMP9 near residues 180–200, corresponding to a flexible loop region near the binding pocket.

Results from RMSD and Rg were utilized to construct free energy landscapes (Figure 6F), which demonstrated well‐defined low‐energy basins for all complexes. Each system displayed a dominant energy minimum, indicating convergence toward stable conformational states over the simulation trajectory. MM/PBSA calculations revealed favorable binding free energies for all complexes (Figure S1A). The estimated ΔGbind values were −9.97 kcal/mol for CYP3A4, −1.86 kcal/mol for HDAC1, −7.21 kcal/mol for JAK2, −12.28 kcal/mol for MMP9, and −10.38 kcal/mol for PARP1.

Residue decomposition analyses identified key energetic contributors in each complex. CYP3A4 binding (Figure S1B) was driven by PHE108, ARG105, PHE215, and ARG106, whereas HDAC1 interactions (Figure S1C) were dominated by HIS877. JAK2 binding (Figure S1D) contributions were highest from LEU855, VAL863, LEU983, GLY935, and GLY856, and in MMP9 (Figure S1E), HIS226, TYR248, VAL223, ARG249, LEU222, MET247, LEU243, and PRO246 were major contributors. Lastly, PARP1 (Figure S1F) binding involved significant contributions from SER904, TYR896, HIS862, PHE897, GLY863, and LYS903. Together, these trajectories support the formation of dynamically stable trigonelline–protein complexes and corroborate the binding‐site assignments from docking.

## 4 Discussion

Ovarian aging encompasses interrelated mechanisms, including ECM remodeling, chronic low‐grade inflammation, oxidative and mitochondrial stress, genomic instability, and altered steroid metabolism (Bochynska et al. 2025). While diet and lifestyle can influence these pathways, most research on coffee and reproductive health has focused on caffeine and epidemiological endpoints (O'Keefe et al. 2018), leaving the roles of coffee‐derived metabolites ambiguous. Trigonelline, a coffee alkaloid present at dietarily achievable concentrations, has been linked to antioxidant, mitochondrial, and longevity‐related actions (Nguyen et al. 2024), but its contribution to ovarian aging has not been examined. Here, we employed an integrated in silico approach, combining network pharmacology, PPI analysis, single‐cell transcriptomics, molecular docking, and molecular dynamics to generate and prioritize hypotheses on how trigonelline may intersect with ovarian aging pathways.

### 4.1 Network Pharmacology and PPI Analysis Identified Five Core Targets in Ovarian Senescence

MMP9, JAK2, PARP1, HDAC1, and CYP3A4. MMP9 regulates ECM turnover, fibrosis and follicular remodeling (Nikanfar and Amorim 2025). JAK2 mediates cytokine and growth factor signaling relevant to granulosa and stromal functions (O'Shea et al. 2013), whereas PARP1 couples DNA repair to primordial follicle activation (Chen et al. 2023). HDAC1 maintains chromatin and mitochondrial stress responses (Shao et al. 2020), and CYP3A4, although primarily hepatic, catalyzes estradiol hydroxylation and steroid clearance (Yu et al. 2005). KEGG, GO, and Reactome enrichment mapped these targets to three mechanistic axes—ECM–inflammatory signaling (MMP9 and JAK2), genome–epigenome stress (PARP1 and HDAC1), and endocrine–xenobiotic metabolism (CYP3A4)—establishing a framework for structural and transcriptomic analyses. Although several of these genes have been associated with follicle loss, stromal fibrosis or altered steroidogenesis, they have not been previously regarded collectively as potential mediators connecting a food‐derived bioactive such as trigonelline to ovarian aging. Future work may compare these trigonelline‐sensitive hubs with targets of other coffee constituents, such as chlorogenic acids, to determine whether effects are trigonelline‐specific or reflect broader coffee‐derived signatures.

Within the ECM–inflammation axis, MMP9 and JAK2 have complementary roles. Goldman (2004) highlights MMP9 activity in theca and stromal compartments during follicular development and luteal regression, whereas Bochynska et al. (2025) report age‐related ovarian fibrosis with increased stromal collagen. Alongside enrichment for collagen catabolic and ECM‐organization processes in our dataset (Mercy Sylus et al. 2020), this is consistent with a model in which age‐related ECM stiffening perturbs MMP9‐dependent remodeling and contributes to ovarian fibrosis, a hypothesis that requires targeted MMP9 activity assays in aged ovarian models. Within the same axis, JAK2 enrichment in PI3K‐Akt and antioxidant pathways suggests that trigonelline could modulate redox resilience via JAK2‐PI3K‐Akt‐NRF2 crosstalk (Nguyen et al. 2024; Liu et al. 2023), which can be tested using NRF2 reporter assays and steroidogenesis readouts in trigonelline‐treated granulosa or stromal cells under oxidative stress, in the context of JAK2's established role in cytokine signaling (O'Shea et al. 2013; Smith et al. 2012).

The genome‐epigenome stress module centers on PARP1 and HDAC1. Both clustered in pathways related to protein deacetylation, chromatin remodeling, mitochondrial function, and oxidoreductase processes, consistent with a role in integrating genomic maintenance with metabolic status. PARP1 coordinates base excision repair and, when dysregulated or depleted with age, can promote ER‐stress‐dependent activation of primordial follicles and compromise genome maintenance (Chen et al. 2023; Shilovsky et al. 2017). HDAC1 shapes histone marks and chromatin accessibility, influencing granulosa cell survival and mitochondrial stress responses (Shao et al. 2020; Ma et al. 2012). Both enzymes utilize NAD^+^/NADH (Mendelsohn and Larrick 2017) and zinc ions (Vogelauer et al. 2012), rendering them sensitive to metabolic and redox fluctuations. Trigonelline's putative action as an NAD^+^ precursor (Membrez et al. 2024) raises a hypothesis that it could indirectly support DNA repair and chromatin stability via shifts in NAD^+^‐dependent enzyme activity. Whether trigonelline alters ovarian NAD^+^ levels or modulates PARP1/HDAC1 in vivo remains unknown and should be tested using NAD^+^ quantification, DNA damage assays and histone acetylation profiling in trigonelline‐treated ovarian cells.

The endocrine–xenobiotic axis is represented mainly by CYP3A4, a key enzyme in steroid hormone biosynthesis, xenobiotic metabolism, and mitochondrial oxidoreductase processes. CYP3A4 catalyzes estradiol 2‐hydroxylation and shapes estrogen metabolite patterns (Yu et al. 2005; Tsuchiya et al. 2005), and may also regulate endothelial prostacyclin production and vasodilation (Jobe et al. 2013). In keeping with this predominantly hepatic role, CYP3A4 transcripts were essentially absent from ovarian single‐cell clusters in GSE255690 and are reported at comparatively low levels in ovarian single cell profiles. These patterns argue against CYP3A4 as a major intragonadal target of trigonelline. Instead, any trigonelline–CYP3A4 interaction is most likely to occur in hepatic or vascular compartments, modifying systemic estrogen metabolite profiles and vascular tone and thereby indirectly influencing ovarian function. Within this framework, CYP3A4 is best conceptualized as a systemic gatekeeper of steroid and xenobiotic metabolism rather than an ovary‐intrinsic effector.

Single‐cell transcriptomic profiling (GSE255690) provided a cell‐type‐resolved view of age‐related remodeling of these targets, as detailed in Table 1. HDAC1 showed strong, widespread expression in young ovaries and declined consistently across multiple cell types in middle‐aged samples, consistent with its role as an ovary‐intrinsic correlate of aging and prior links between reduced histone deacetylase activity, epigenetic drift and heightened stress susceptibility (Ozturk 2025). PARP1 was modestly expressed across most lineages, exhibiting small but significant age‐associated differences, with generally lower expression in middle‐aged stromal and vascular compartments but slightly higher levels in granulosa cells, aligning with its role in DNA damage responses (Shilovsky et al. 2017) and stress adaptation (Chen et al. 2023). MMP9 expression was largely confined to monocytes, where it was strongly upregulated with age, and to theca‐stroma cells, where it had low‐level expression, suggesting niche‐specific functions in ECM turnover and fibrosis (Goldman 2004). JAK2 displayed heterogeneous expression, decreasing in several stromal and endothelial populations but increasing in monocytes, reflecting its role as a broad integrator of cytokine and stress signaling (Li et al. 2021). By contrast, CYP3A4 was essentially non‐expressed, reinforcing the view that its contribution to the trigonelline‐ovarian aging axis is likely systemic.

Molecular docking and 100‐ns molecular dynamics simulations provided a structural view of plausible trigonelline–protein interactions. Docking scores indicated moderate affinities for all five targets, consistent with trigonelline's small, rigid, highly polar structure (Nguyen et al. 2024). Because standard docking only approximates protein flexibility and solvation, these scores are best used to rank targets and suggest that trigonelline is unlikely to act as a high‐affinity ligand, while still allowing biologically relevant contacts. Predicted poses mapped to key functional regions—the zinc‐dependent catalytic cleft of MMP9 (Maskos and Bode 2003), the NAD^+^‐binding groove of PARP1 (Langelier et al. 2018), the ATP‐binding hinge of JAK2 (Bandaranayake et al. 2012), and the distal substrate‐binding cavity of CYP3A4 (Sevrioukova 2022) – while binding to HDAC1 was predicted adjacent to the catalytic channel (Zhu et al. 2010), with interpretation limited by noncanonical residue numbering and model uncertainty. MD trajectories supported stable, compact complexes without major unfolding, and local flexibility patterns, including a mobile loop in MMP9, were consistent with known metalloproteinase dynamics (Maskos and Bode 2003). MM/PBSA, an approximate endpoint method that neglects full entropic and solvent effects (Genheden and Ryde 2015), yielded favorable binding free energies, ranking MMP9 and PARP1 highest, followed by CYP3A4 and JAK2, and substantially weaker binding for HDAC1. These values are therefore best treated as qualitative rankings rather than precise affinities, and weaker or ambiguous docking/MD results should motivate down‐prioritizing, rather than excluding, particular targets as direct trigonelline binders, since modest interactions could still support indirect or context‐dependent modulation in vivo.

Integrating these data, this framework supports a hierarchical view of trigonelline‐responsive targets in ovarian aging. PARP1 and MMP9 emerge as the strongest candidates, with convergent support from network centrality, pathway enrichment, structural modeling, and age‐sensitive expression in relevant cell types. Trigonelline's potential to influence NAD^+^‐dependent PARP1 activity (Mendelsohn and Larrick 2017; Membrez et al. 2024) and ECM‐remodeling MMP9 activity (Goldman 2004; Raffetto and Khalil 2008) provides a coherent hypothesis linking redox, genome maintenance, inflammation and fibrosis in ovarian aging. JAK2 and HDAC1 also appear mechanistically relevant, but current structural data favor indirect or context‐dependent modulation rather than strong direct binding, while CYP3A4 is most plausibly a systemic mediator acting through hepatic estrogen metabolism and vascular tone, consistent with its role as a gatekeeper of circulating steroid and xenobiotic metabolism rather than an ovary‐intrinsic target (Yu et al. 2005; Tsuchiya et al. 2005; Jobe et al. 2013).

Several of the hubs highlighted here are already targeted by approved drugs or late‐stage clinical candidates, underscoring the pharmacological accessibility of these pathways. PARP1 is inhibited by PARP inhibitors such as olaparib and related agents used as maintenance therapy in ovarian and other homologous recombination–deficient cancers (Moore et al. 2018; Ledermann and Pujade‐Lauraine 2019). JAK2 serves as a principal target of JAK1/2 inhibitors such as ruxolitinib and momelotinib, which are approved for myelofibrosis and other myeloproliferative neoplasms (Masarova and Chifotides 2025). HDAC1 belongs to the class I HDAC family modulated by broad‐spectrum HDAC inhibitors such as vorinostat and romidepsin, which are employed in hematologic malignancies including cutaneous T‐cell lymphoma (Bates 2020). Collectively, these examples indicate that the signaling axes engaged by trigonelline sit within an established druggable space, suggesting potential synergies but also possible interactions that warrant consideration when designing nutritional interventions in individuals receiving such therapies.

Several limitations temper these conclusions. All findings derive from in silico analyses based on public datasets and structural models, and should therefore be regarded as hypothesis‐generating rather than causal. Trigonelline concentrations in ovarian and follicular fluid remain unmeasured, and the low micromolar plasma levels typically achieved after coffee intake constrain inference about dose and exposure windows. Moreover, the simulation methods sample a limited subset of protein conformations and simplified molecular environments, and the single‐cell RNA‐seq dataset represents a relatively small, geographically restricted donor cohort with cross‐sectional sampling and incomplete control of environmental and metabolic covariates such as BMI, smoking, diet, and systemic inflammation. Inter‐individual variation in coffee intake patterns and trigonelline metabolism across populations may further limit generalizability. As a result, both binding predictions and age‐associated expression patterns should be considered preliminary and reevaluated as more diverse cohorts and higher‐resolution experimental and omics data become available.

Despite these caveats, the present work defines a concrete experimental agenda. Immediate priorities include examining whether physiologically relevant trigonelline exposures modulate PARP1‐dependent DNA repair and follicle survival (e.g., PARPylation, DNA damage markers) and MMP9‐driven ECM remodeling (e.g., collagen content) in ovarian and granulosa models. Furthermore, JAK2‐NRF2 signaling, NAD + ‐linked epigenetic pathways involving HDAC1 and CYP3A4‐dependent estrogen metabolite profiles in hepatic or vascular models represent complementary lines of inquiry. From a nutritional perspective, defining dose–response relationships, tissue exposure and long‐term safety under habitual coffee consumption will be essential to translate these mechanisms into dietary strategies for ovarian aging.

## 5 Conclusion

This integrative in silico study suggests that the coffee‐derived alkaloid trigonelline may influence ovarian aging by converging on a limited set of targets and pathways, with PARP1‐centered genome maintenance and MMP9‐mediated extracellular matrix remodeling emerging as the most credible hubs, and JAK2, HDAC1, and CYP3A4 providing additional signaling, epigenetic, and endocrine‐xenobiotic links. By combining network pharmacology, molecular simulations, and single‐cell transcriptomic analysis, we narrow the mechanistic gap between trigonelline's known systemic bioactivities and ovarian aging biology and prioritize specific genes and pathways for experimental testing. These findings are hypothesis‐generating and require validation in ovarian cell models, animal studies, and ultimately, dietary or supplementation studies. Nevertheless, they support further evaluation of trigonelline as a coffee‐derived candidate for functional foods or nutraceutical strategies targeting reproductive aging.

## Author Contributions

**Woon Shin Yong:** writing – original draft, visualization, data curation, formal analysis, investigation, methodology. **Yuankun Han:** methodology, writing – review and editing, formal analysis, visualization. **Yulu Shen:** writing – review and editing, investigation, formal analysis, visualization. **Ningyu Sun:** writing – review and editing, conceptualization, methodology, formal analysis, supervision. **Qinhua Zhang:** conceptualization, supervision, project administration, funding acquistion, writing – review and editing.

## Ethics Statement

This study did not involve human or animal experiments and therefore did not require institutional ethics approval.

## Consent

The authors have nothing to report.

## Conflicts of Interest

The authors declare no conflicts of interest.

## Supporting information


**Data S1:** Supporting Information.

## Data Availability

All data supporting the conclusions of this study are included in this article. Additional information or underlying datasets can be obtained from the corresponding author upon reasonable request.
