# Supplementary material for: From Coffee Alkaloid to Ovarian Targets: An Integrated Computational Framework for Trigonelline in Ovarian Aging
Source: Food Sci Nutr. 2026 Feb 10;14(2):e71421. doi: 10.1002/fsn3.71421 (PMC12887449; doi:10.1002/fsn3.71421)
Supplement: Supplementary file 1 — Data S1: Supporting Information. [file FSN3-14-e71421-s001.docx]

**Supplementary Material**

**Supplementary Table S1. Centrality metrics for top-ranked nodes in the STRING PPI network of trigonelline-ovarian aging targets.**

| No. | Gene | Degree | MNC | MCC | Betweenness |
| --- | --- | --- | --- | --- | --- |
| 1 | MMP9 | 18 | 18 | 102 | 387.92 |
| 2 | PARP1 | 15 | 14 | 67 | 453.48 |
| 3 | JAK2 | 13 | 13 | 78 | 261.91 |
| 4 | HDAC1 | 12 | 9 | 43 | 504.80 |
| 5 | CYP3A4 | 10 | 9 | 43 | 284.89 |
| 6 | MMP2 | 10 | 10 | 50 | N/A |
| 7 | NOS3 | 9 | N/A | N/A | N/A |
| 8 | CYP19A1 | 9 | N/A | 62 | 241.23 |
| 9 | SCARB1 | 9 | 8 | N/A | N/A |
| 10 | MAOA | N/A | N/A | N/A | 359.40 |
| 11 | KIT | N/A | 8 | N/A | N/A |
| 12 | CCR2 | N/A | 8 | N/A | N/A |
| 13 | CYP17A1 | N/A | 8 | 56 | N/A |
| 14 | HSD11B1 | N/A | N/A | 56 | N/A |
| 15 | CYP11B1 | N/A | N/A | 48 | N/A |
| 16 | ACHE | N/A | N/A | N/A | 133.74 |
| 17 | NOS2 | N/A | N/A | N/A | 144.67 |
| 18 | SIGMAR1 | N/A | N/A | N/A | 179.00 |

**Abbreviations:** MNC, maximum neighborhood component; MCC, maximal clique centrality; N/A, not in the top-10 list for that centrality metric.


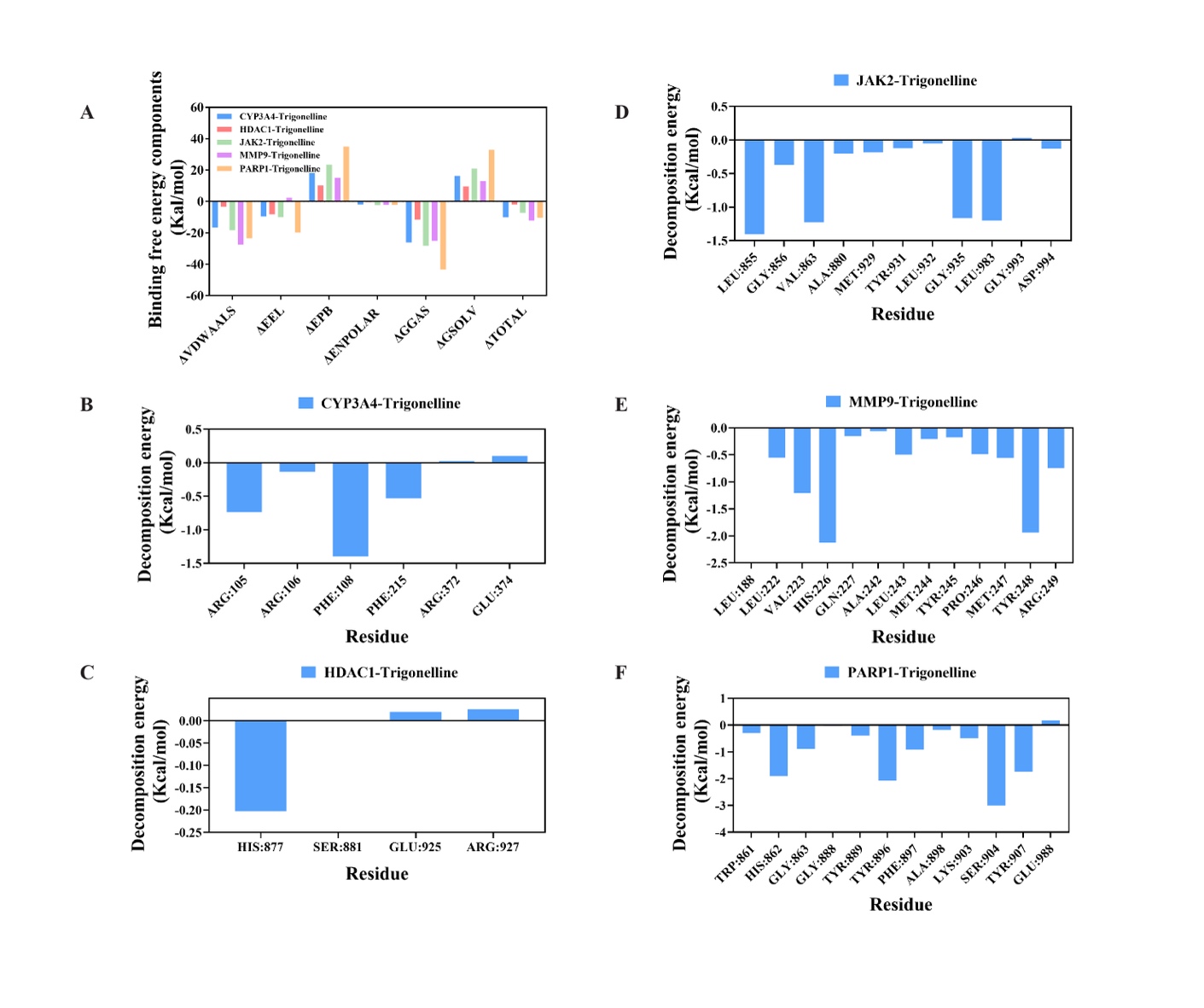
**Supplementary Figure S1. MM/PBSA analysis of trigonelline binding to CYP3A4, HDAC1, JAK2, MMP9, and PARP1.**
(A) Predicted binding free energies (ΔG_bind) for each complex, showing favorable binding for all targets, with the most negative values for MMP9 and PARP1, intermediate values for CYP3A4 and JAK2, and weaker binding for HDAC1.
(B–F) Per-residue binding energy decomposition for CYP3A4 (B), HDAC1 (C), JAK2 (D), MMP9 (E) and PARP1 (F), highlighting key amino acids that contribute most strongly to trigonelline binding and overlap with known functional pocket residues.
